# Sphincter saving anorectoplasty (SSARP) for the reconstruction of Anorectal malformations

**DOI:** 10.1186/1471-2482-7-20

**Published:** 2007-09-24

**Authors:** Akshay Pratap, Awadhesh Tiwari, Anand Kumar, Shailesh Adhikary, Satyendra Narayan Singh, Bishnu Hari Paudel, Rajiv Bartaula, Brijesh Mishra

**Affiliations:** 1 Division of Pediatric Surgery, Department of Surgery, B.P. Koirala Institute of Health Sciences, Dharan, Nepal; 2 Department of Radiology, B.P. Koirala Institute of Health Sciences, Dharan, Nepal; 3 Department of Anesthesia, B.P. Koirala Institute of Health Sciences, Dharan, Nepal; 4 Department of Physiology, B.P. Koirala Institute of Health Sciences, Dharan, Nepal

## Abstract

**Background:**

This report describes a new technique of sphincter saving anorectoplasty (SSARP) for the repair of anorectal malformations (ARM).

**Methods:**

Twenty six males with high ARM were treated with SSARP. Preoperative localization of the center of the muscle complex is facilitated using real time sonography and computed tomography. A soft guide wire is inserted under image control which serves as the route for final pull through of bowel. The operative technique consists of a subcoccygeal approach to dissect the blind rectal pouch. The separation of the rectum from the fistulous communication followed by pull through of the bowel is performed through the same incision. The skin or the levators in the midline posteriorly are not divided. Postoperative anorectal function as assessed by clinical Wingspread scoring was judged as excellent, good, fair and poor. Older patients were examined for sensations of touch, pain, heat and cold in the circumanal skin and the perineum. Electromyography (EMG) was done to assess preoperative and postoperative integrity of external anal sphincter (EAS).

**Results:**

The patients were separated in 2 groups. The first group, Group I (n = 10), were newborns in whom SSARP was performed as a primary procedure. The second group, Group II (n = 16), were children who underwent an initial colostomy followed by delayed SSARP. There were no operative complications. The follow up ranged from 4 months to 18 months. Group I patients have symmetric anal contraction to stimulation and strong squeeze on digital rectal examination with an average number of bowel movements per day was 3–5. In group II the rate of excellent and good scores was 81% (13/16). All patients have an appropriate size anus and regular bowel actions. There has been no rectal prolapse, or anal stricture. EAS activity and perineal proprioception were preserved postoperatively. Follow up computed tomogram showed central placement the pull through bowel in between the muscle complex.

**Conclusion:**

The technique of SSARP allows safe and anatomical reconstruction in a significant proportion of patients with ARM's without the need to divide the levator plate and muscle complex. It preserves all the components contributing to superior faecal continence, and avoids the potential complications associated with the open posterior sagittal approach.

## Background

Posterior sagittal anorectoplasty (PSARP), popularized by de Vries and Peña has become the preferred technique for surgical management of anorectal malformations (ARM) [1]. The PSARP involves incision from coccyx to perineal body, to widely expose the external sphincter, the levators, the rectum, and distal fistula to facilitate surgical repair. Dividing the sphincter posteriorly can affect the pudendal nerve and its terminal branches to the sphincter in these patients, who already may be having widespread lumbar and sacral lesions [2]. Despite excellent exposure of the anatomy and exact placement of the distal rectum within the muscle complex, continence often is less than ideal [3,4]. In an attempt to preserve the neurophysiological function of the sphincter we describe a sphincter saving approach to reconstruct ARM.

## Methods

Between March 2005 and May 2007, 26 children have undergone a sphincter saving anorectoplasty (SSARP) for high ARM. This study was conducted in accordance with the principles of the Declaration of Helsinki and 'good clinical practice' guidelines. The protocol was approved by the Ethical Review Board of B.P. Koirala Institute of Health Sciences (Ethical Review Board number: 497/062/063). Prior to the surgery written informed consent was obtained from the parents of children.

### Exclusion criteria

Patients with cloacal malformations, rectovesical fistula, rectovestibular fistula and low ARM were excluded from the study. Hospital charts and surgical notes were reviewed and clinical characteristics tabulated.

### Preoperative management

A thorough perineal examination, urine analysis and an invertogram was done to distinguish between high and low anorectal defects. An echocardiogram was done to evaluate congenital heart defects.

### Invertography

**Figure 1:**
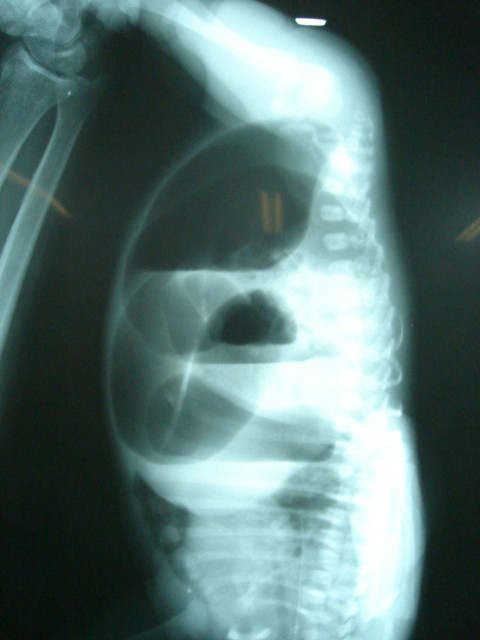
Invertogram showing a high blind pouch in a newborn undergoing primary SSARP. A blind distal pouch within 1 cms from the last vertebral bone can be corrected by this approach.

This examination was performed at least 12 – 18 hours after delivery in all newborns, using the method described by Wangensteen and Rice [5]. The lateral radiogram was done in the inverted position, with the radio-opaque marker fixed in the would-be-anus site to measure the distance between the most distal gas collection and skin and the last ossified vertebral bone (Figure 1).

### Preoperative electromyography of external anal sphincter

Patients older than 3 years were subjected to preoperative electromyography (EMG) of the external anal sphincter (EAS). Electrical activity was evaluated by needle electrodes in all the patients. A disposable 37 mm standard concentric needle electrode was inserted into the EAS as described by Podnar et al, to evaluate both the superficial part and deep parts of the muscle [6]. The subcutaneous part of the EAS was evaluated by placing the needle electrode perpendicularly to the anoderm at a depth of a few millimeters at the site of anal dimple.

### Preoperative localization of muscle complex

**Figure 2:**
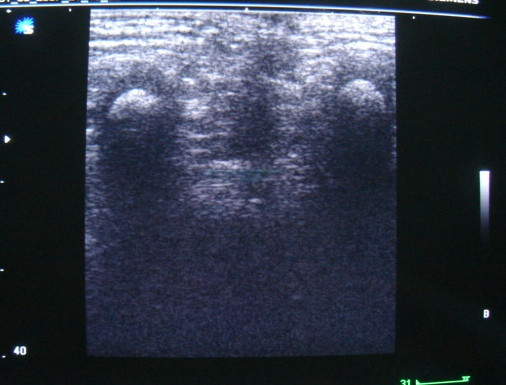
Preoperative localization of the center of muscle complex. A perineal sonogram showing muscle complex on two sides with variable amount of levator fat.

**Figure 3:**
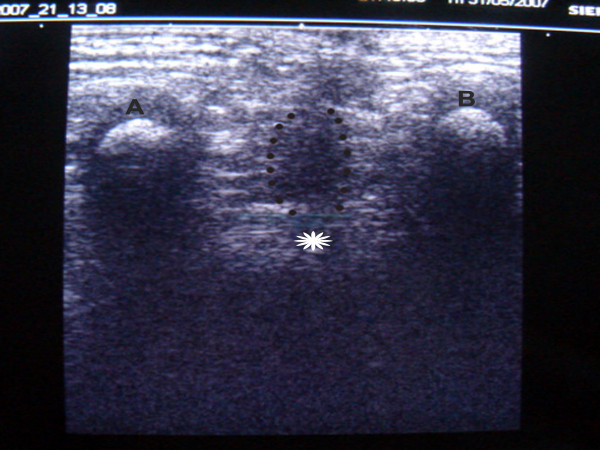
Sonogram showing important anatomical landmarks which guide accurate central placement of guide wire. Points A and B indicate ischial tuborosities. Dashed curved line indicates the medial borders of the levators on both sides. The star in the center corresponds to the position of the anal dimple.

**Figure 4:**
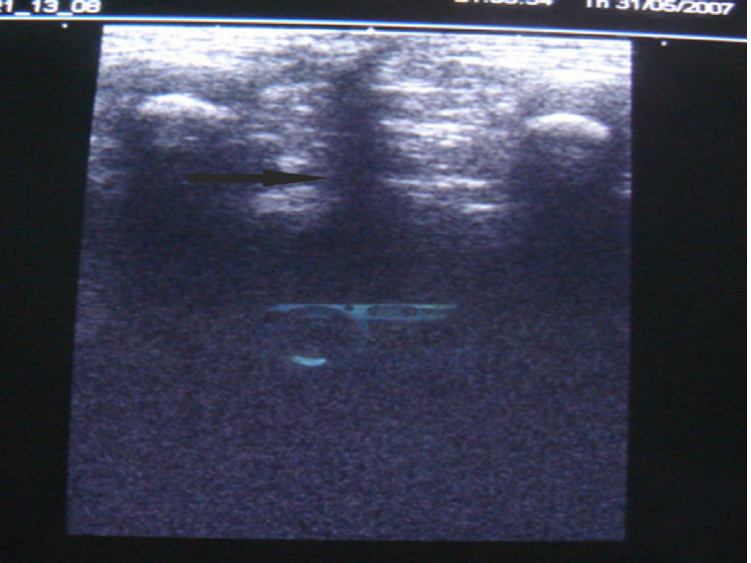
Sonogram showing guide wire (arrow) in the center of the muscle complex.

**Figure 5:**
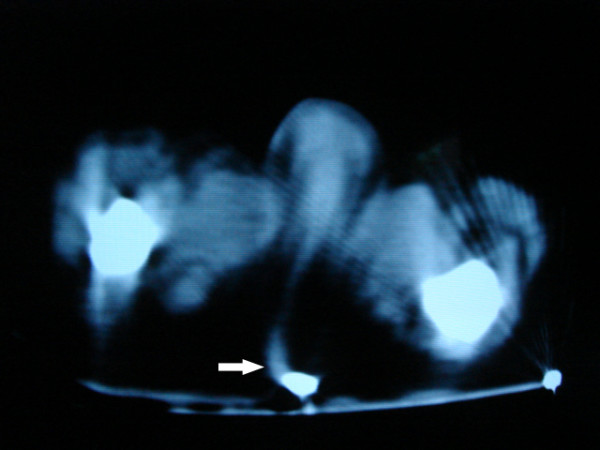
A computed tomogram verifying the central placement of the guide wire (arrow).

To facilitate accurate central placement of bowel within the muscle complex without having to divide the levators all patients underwent real time sonography of the perineum. The child was placed in a prone position with elevated pelvis and legs and perineal anatomy studied a linear 5 MHz probe  (Figure 2). The two ischial tuborosities and the medial borders of muscle complex on either side serve as landmarks to ensure central placement of the cannula (Figure 3). The center of the external sphincter on the anal dimple is marked using a muscle stimulator (Bajpai, [20]). A 16 gauge cannula is advanced through the center of the external sphincter till it lies just below the levators as seen on a transverse real time scan. Once the correct depth of the tip of the cannula is verified, it is advanced in the midline towards the coccyx under the guidance of longitudinal real time scan (Figure 4). The cannula is then brought out just below the last palpable vertebral bone. A soft guide wire is pushed over the cannula and secured. The correct placement of the guide wire is checked on a CT scan (Figure 5) with parameters settings adjusted based on the size of the child and region of interest [7]. The path of the guide wire is then dilated during surgery which serves as a tunnel for pull through bowel.

### Augmented-pressure distal cologram

This examination was done on children with previously constructed colostomy. A lateral radiogram of the pelvis was made after injecting the contrast medium via a Foley catheter into the distal bowel with sealing of the distal stoma [8].

### Primary or staged SSARP

The decision to perform a primary SSARP was determined by the neonate's general condition, and the presence of the air column within the rectum blind pouch within 1 cms of the last ossified vertebral bone (Figure 1). If the baby was unwell with features of sepsis or a very high pouch a high sigmoid colostomy was made and SSARP performed at a later date.

### Surgical technique

**Figure 6:**
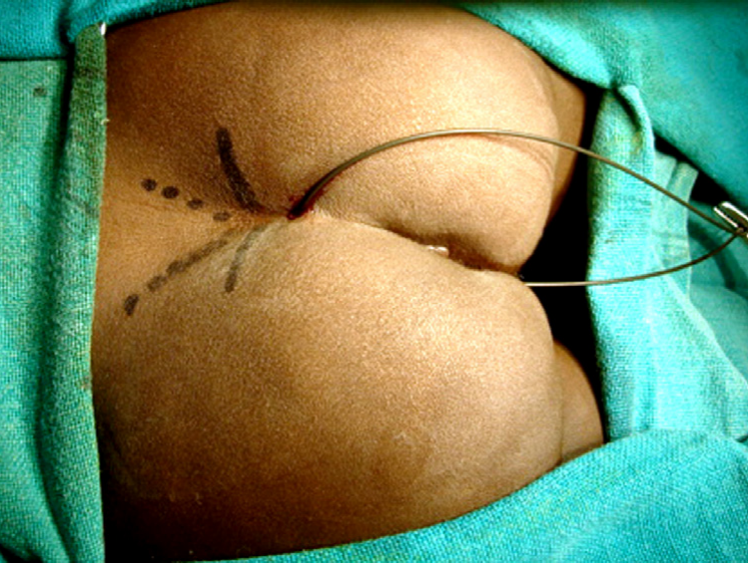
A transverse incision is made over the last palpable vertebra.

**Figure 7:**
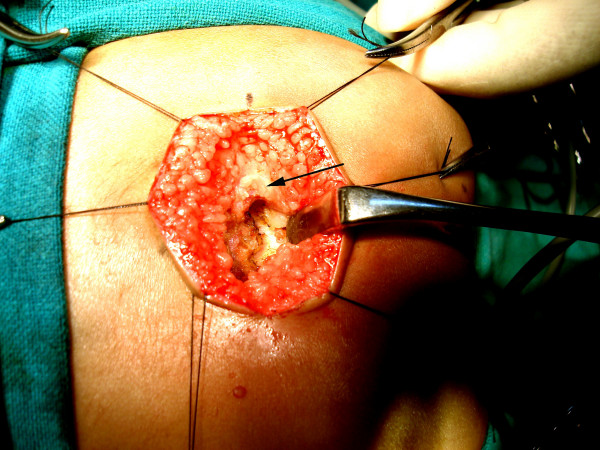
Dissection is deepened to expose the coccyx. The periosteum over the coccyx is removed and coccygectomy performed (arrow).

**Figure 8:**
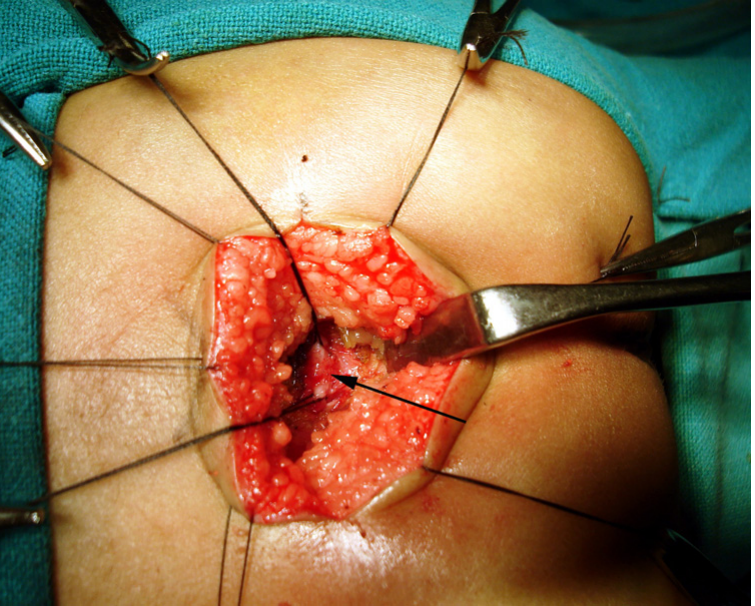
The blind rectal pouch is isolated (arrow).

**Figure 9:**
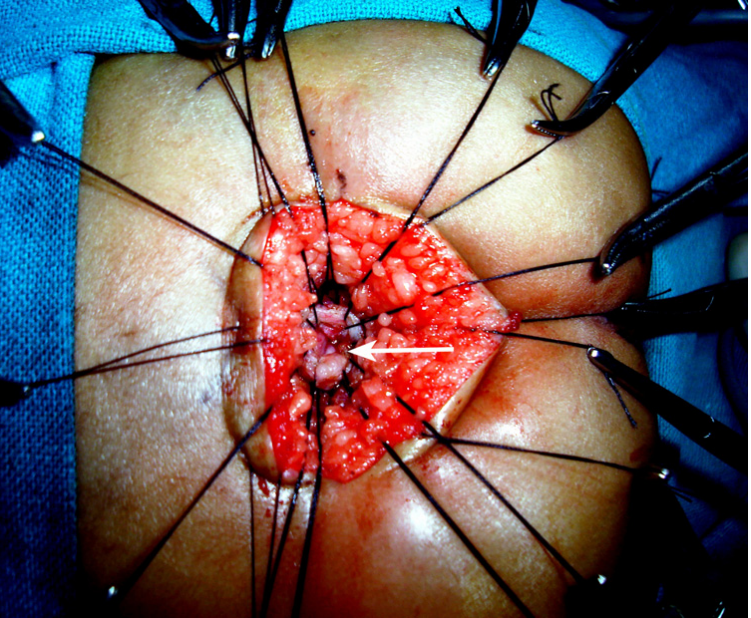
The rectal pouch is opened longitudinally (arrow).

**Figure 10:**
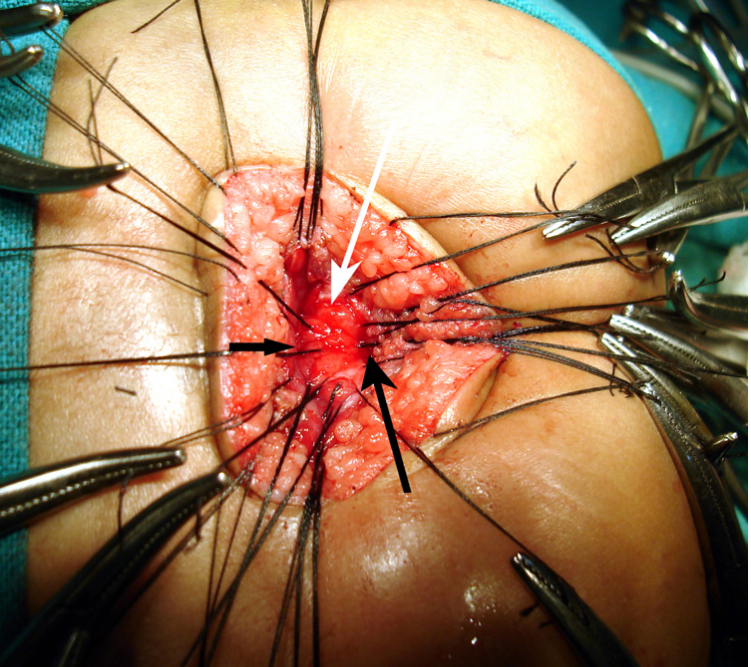
The separation of the rectum (black small arrow) and the urethra is started by creating a plane of dissection in the common wall of the fistula (white arrow).

**Figure 11:**
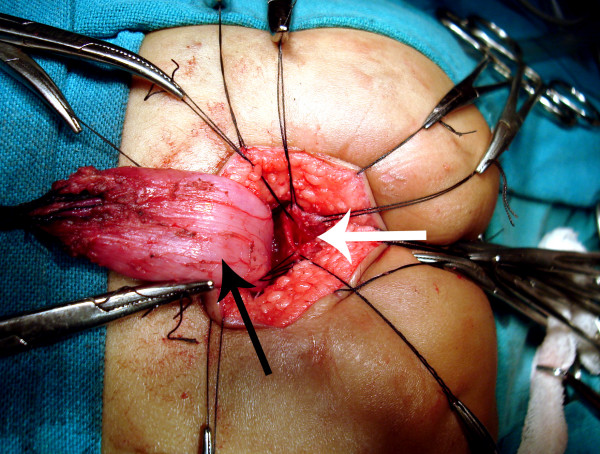
Complete separation of the rectum (black arrow) from the fistula (white arrow) continues until the fistula is completely free.

**Figure 12:**
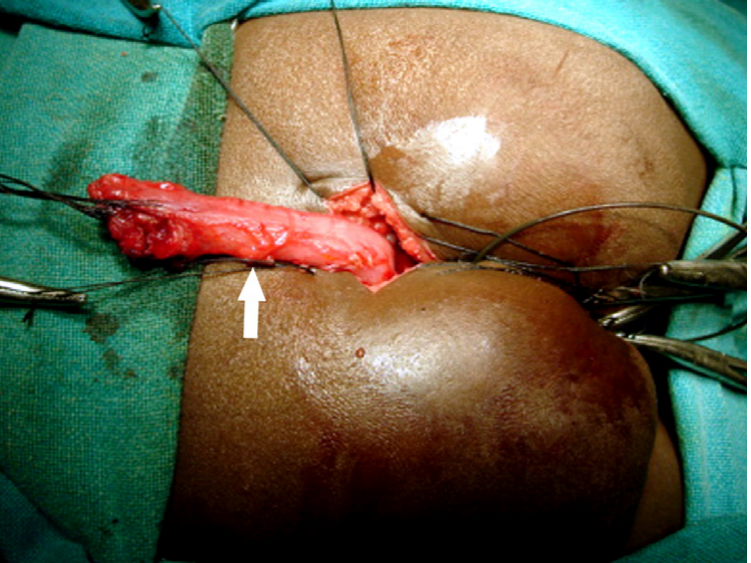
Rectum (arrow) is mobilized upwards by clearing the perirectal adhesions so that adequate length is available for pull through without anastomotic tension.

**Figure 13:**
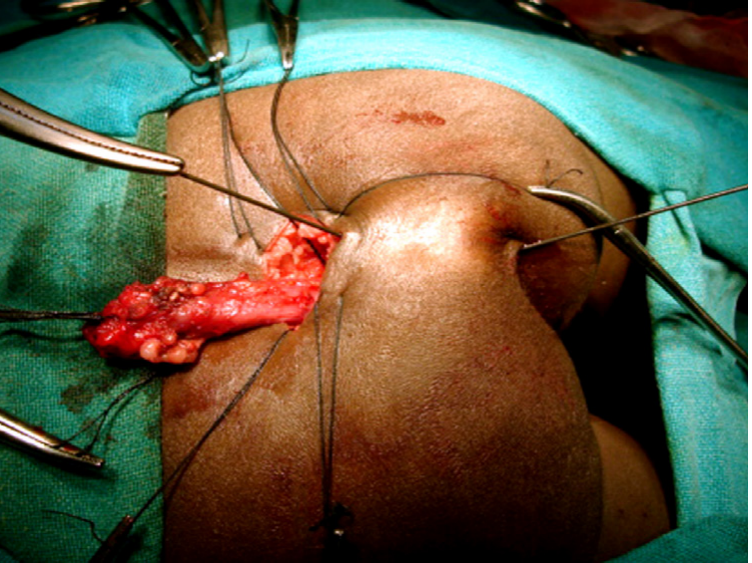
The path of guide wire is dilated serially till it accommodates a appropriate sizes Hegar Dilator.

**Figure 14:**
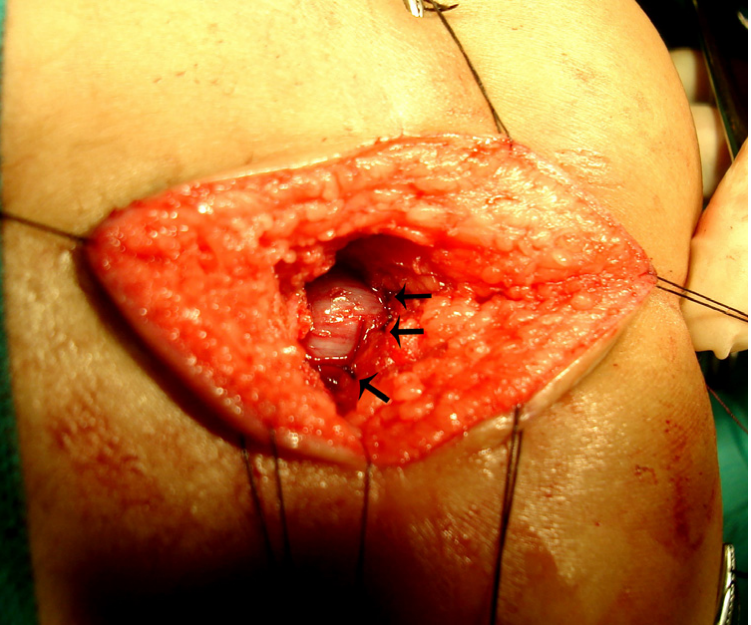
Rectal pouch is fixed to the proximal edge of levator plate by several non absorbable sutures (small arrows).

**Figure 15:**
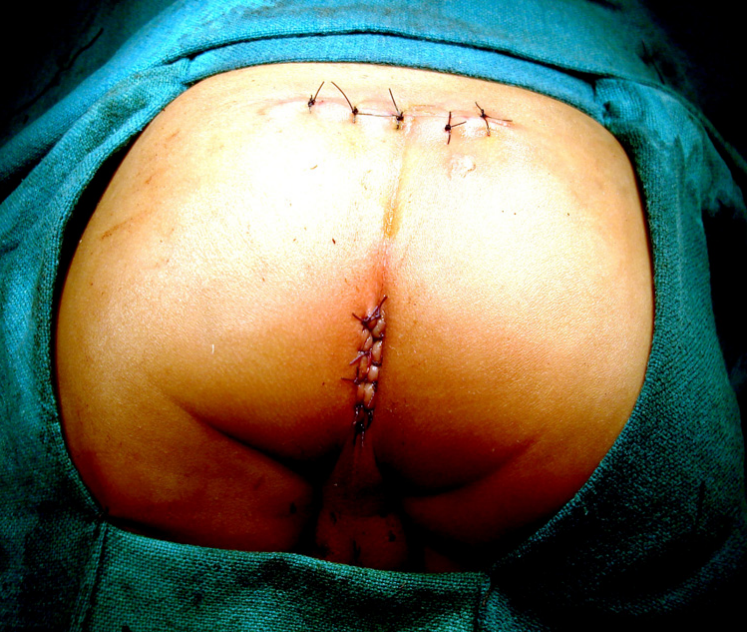
At completion of SSARP. Note the complete sparing of the posterior midline skin and excellent cosmetic appearance.

After the bladder is catheterized, the patient is placed in prone jackknife position (Figure 6). A transverse incision is made just above the coccyx (Figure 7). In patients with sacral agenesis the incision is made over the last palpable spine. The incision is deepened in its subcutaneous plane to expose the coccyx. The periosteum over the coccyx is removed and coccygectomy performed (Figure 8). No dissection is done in the midline. The rectal blind pouch is mobilized within Waldeyer's fascia. The rectal pouch is detached from the anterior area of the sacrum by using a finger. Laterally the rectal pouch is attached to the pelvis by a thick ligament with the middle haemorrhoidal artery. Resection of this ligament is necessary for detaching the rectal pouch. The blind rectal pouch is opened longitudinally (Figure 9). Separation of the rectal pouch from the urethra or the vagina is performed by combined sharp and blunt dissection. The separation of the rectum and the urethra is started by creating a plane of dissection in the common wall of the fistula (Figure 10). Once a plane is found, the separation of the rectum from the fistula continues until the fistula is completely free (Figure 11). The fistula is closed in two layers. Rectum is mobilized upwards by clearing the perirectal adhesions so that adequate length is available for pull through without anastomotic tension (Figure 12). The external sphincter is once again defined with electrostimulation. The path of guide wire which was placed preoperatively is serially dilated until it accommodates Hegar dilators of sizes 6 – 12 (Figure 13). The rectum is then pulled down through the tunnel and fixed to the proximal edge of levator plate by several sutures (Figure 14). Finally a standard anorectoplasty performed (Figure 15). The transverse incision is closed in layers.

### Postoperative management

Two weeks after the repair, the patient was started on a protocol of anal dilatations. If a colostomy was constructed it was closed after the neoanus accepted the desired size of dilator.

### Assessment of continence

At the follow-up visit, modified Wingspread Scoring [9] was adopted to investigate the fecal condition in children older than 3 years. The operative outcome was designated as "excellent," "good," "fair," and "poor." Continence was defined as the ability to stay clean without staining or soiling both day and night without pads or diapers. Soiling was defined as an inadvertent loss of small amounts of feces staining the underwear. Incontinence was defined as regular loss of solid feces. Constipation was defined as less than 3 spontaneous bowel movement per week, rectal impaction, or abdominal fecalomas.

### Post operative electromyography of external sphincter

Post operative EMG was performed 3 weeks after anorectoplasty. Any sphincter abnormality was assessed by investigating the location, integrity and activity of EAS. Activity at rest and under voluntary contraction were analyzed and compared to their preoperative values.

## Results

Ages, sexes, as well as other main features of the patients who underwent surgery with this technique are summarized in Table 1. In 10 newborns, SSARP was performed as a primary procedure without prior colostomy. Sixteen children were treated with initial colostomy in the newborn period followed by delayed SSARP. There was no operative mortality. There was no difficulty in gaining enough length for pull-through, regardless of whether the patients had undergone prior colostomy. No conversion to traditional PSARP approach was necessary. The mean operating time was 72 minutes ± 13.2. The follow-up ranged from 4 months to 22 months. No patients have shown ischemia or stricture of the anorectal anastomosis. We obtained excellent results with regards to short hospital stay, functional bowel movements and attractive cosmetic results.

**Table 1:** Patient Characteristics and types of anomalies

Patient No.	Age at SSARP	Fistula	Associated anomaly	Follow up (months)
1	40 h	Rectoprostatic	VUR	13
2	38 h	Rectoprostatic	Agenesis of left kidney	12
3	55 h	Rectoprostatic	VUR	15
*4	36 mo	Rectobulbar	none	6
*5	44 mo	Rectoprostatic	Proximal hypospadias	12
6	26 h	Rectoprostatic	Trisomy 21	13
7	32 h	Rectoprostatic	none	10
*8	44 mo	Rectoprostatic	none	18
9	28 h	Rectoprostatic	ASD	15
*10	73 mo	Rectoprostatic	none	14
11	30 h	Rectobulbar	none	9
*12	40 mo	Rectobulbar	none	6
*13	66 mo	Rectobulbar	Sacral hemi vertebra	4
*14	72 mo	Rectoprostatic	VUR	5
15	72 h	Rectoprostatic	Midpenile hypospadias	11
*16	56 mo	Rectoprostatic	none	16
17	55 h	Rectoprostatic	VACTERL	13
*18	33 mo	Rectoprostatic	none	11
*19	48 mo	Rectoprostatic	none	10
*20	39 mo	Rectoprostatic	none	6
21	36 h	Rectoprostatic	none	6
*22	39 mo	Rectoprostatic	Sacral hemi vertebra	20
*23	52 mo	No fistula	none	15
*24	55 mo	No fistula	Sacral hemi vertebra	12
*25	37 mo	Rectoprostatic	none	14
*26	48 mo	Rectoprostatic	none	4

*Sigmoid colostomy was performed prior to SSARP.

### Bowel function in neonates (Group I)

The 10 neonates and infants, who are yet too young to be evaluated for continence, have symmetric anal contraction to stimulation and strong squeeze on digital rectal examination. The average number of bowel movements per day was 3–5, without the need for any laxative or enema.

### Bowel function in older children (Group II)

The functional outcome was assessed in 16 of our patients who are now older than 3 years of age. Fecal continence was excellent and good in 13 (81%) and fair and poor in 3 (19%) of the patients, Table 2. The average number of bowel movements was 2–5 per day.

**Table 2:** Results according to Clinical, Radiological and Electromyography assessment

**Parameters for assessment of anorectal function**		**Group II N = 16**
Clinical scoring	Excellent and good	13(81%)
	Fair and Poor	3(19%)
Sensation of perineum and anal canal after surgery	Cold	16(100%)
	Heat	16(100%)
	Pain	16(100%)
External anal sphincter integrity on EMG	Preserved	13(81%)
	Disrupted	3(19%)
CT pelvis for placement of bowel	Correctly placed	16(100)
	Misplaced	0

### Perineal sensations

Preoperative perception of heat cold and painful stimuli was present in all 16 children. These children continued to appreciate the sensations with the same magnitude in the postoperative period.

### Follow up Computed Tomography

**Figure 16:**
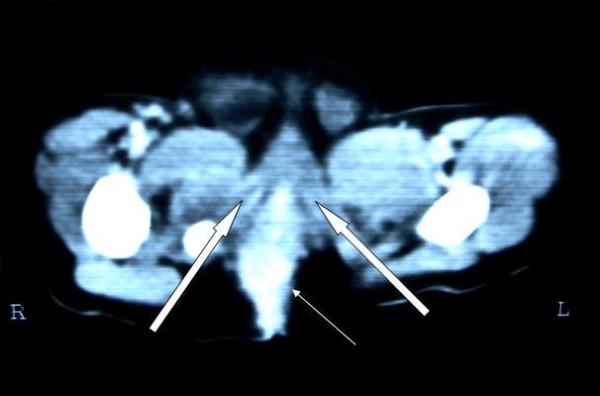
Follow up CT scan showing rectum (straight arrow) placed in midline to the muscle complex (block arrows).

Follow up CT scans revealed rectum placed in the center of the muscle complex in all patients (Figure 16).

### Electromyographic studies of EAS

**Figure 17:**
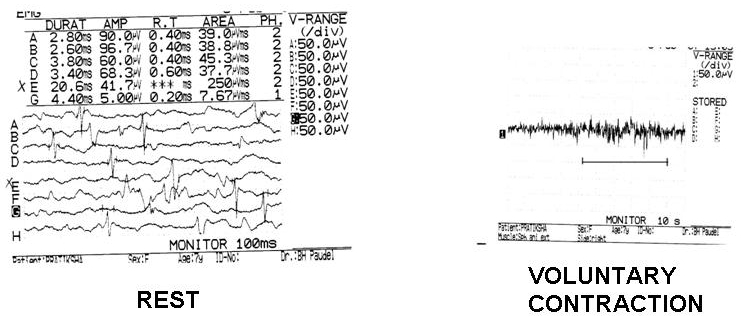
Postoperative EMG of EAS during rest and voluntary contraction showing preservation of integrity and function of EAS showing polyphasic motor unit potentials (scale).

Preoperative electrical activity of the EAS was seen in 13 patients, which was preserved after SSARP (Figure 17). Three patients with absent EAS activity had sacral agenesis.

## Discussion

Despite large research and improved understanding of embryology and pathophysiology of ARM, the problem of obtaining better functional results still remains unsolved [3,4]. Stephen's anatomical observations with regard to the course of the nervi erigentes were decisive for the development of his sacrococcygeal technique [10]. However, his technique was considered blind, as it did not ensure accurate placement of the pull through bowel in the center of the muscle complex, and fell into oblivion. It is to the credit of Pena and De Vries who introduced the posterior sagittal anorectoplasty (PSARP) that most pediatric surgeons have found it now easy to repair IA [1,11]. Despite performing a technically "perfect" operation, there are a subset of children that require significant lifelong bowel management for constipation or incontinence [12]. These children are often characterized by a very abnormal sacrum, poor perineal musculature, colonic dysmotility, and deficient pelvic innervation. It is not known whether anything can be done to improve the outcome for children with these poor prognostic factors. However, there is substantial evidence that to emphasize the importance of preservation of the mucosal receptors at the level of the internal sphincter, lowermost part of the rectum and the proprioceptors at perineal skin in maintaining continence [13,14]. The sphincter saving anorectoplasty (SSARP) described in this report provides an opportunity to maximize preservation of all existing continence mechanisms in these children. This small series does highlight several advantages of this approach. 1) A transverse incision, well away from the neoanus and sparing the midline skin minimizes surgical site infection.

**Figure 18:**
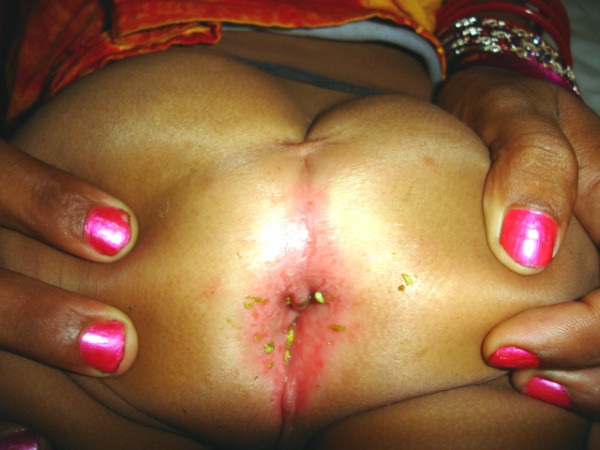
A follow up clinical photograph showing a healthy scar well away from the anus, and a cosmetically normal looking perineum.

2) The skin is not violated in posterior midline sagitally. This preserves the proprioceptive nerves, allowing better sensation which may assist in attainment of continence by providing a "sensory warning zone" [14].

3) Complete preservation of levator muscle ensures integrity of the neurovascular bundle. 4) Central placement of pull through bowel without having to divide the muscle complex in the midline. 5) Finally, the cosmetic appearance of the perineum is satisfactory, resembling the normal surface anatomy (Figure 18). One may argue that dividing the sphincter exactly in the midline, as in PSARP, is relatively safe regarding preservation of the neurovascular bundle, however, violation of this strictly midline approach is common during the "learning curve" for PSARP which may damage the sphincter. Studies have also shown that even cutting the sphincter muscle exactly in the midline was associated with decrease in mean amplitude on EMG of external anal sphincter [15]. Under a technical perspective the only advantage of PSARP is opportunity to directly visualize the muscle complex which assists correct placement of the rectal tube. This aspect is probably the most crucial step in anorectal reconstruction and every effort should be made to correctly place the pull through bowel. SSARP achieves the same goal without having to divide and open the muscle complex. In our study we used CT scan to confirm the correct placement of guide wire preoperatively and the pull through bowel in the postoperative period. Despite the crucial role of a CT scan in this study, the risk of radiation needs to be addressed. Magnetic resonance imaging and intraoperative ultrasound are attractive alternatives to CT scan where these facilities are available. Otherwise strategies to reduce or eliminate unnecessary radiation that children get from CT examinations need to be implemented. These include focused CT examinations by limiting the field of view to the region of interest, and reducing the tube current by 50%. These strategies decrease the radiation dose by 50% without loss of information. Perfect continence is probably not realistic in patients with ARM. The results of clinical evaluation in postoperative patients with anorectal malformations vary depending on the operative methods used by various investigators. In most series continence rates are reported between 8% to 75% and improve with time [3,16]. In our study, sphincter integrity was preserved after SSARP in all the 13 patients who showed EMG activity preoperatively. The remaining three children who had sacral agenesis had no EAS activity on EMG and were found to have fair continence scores. These three children however, have preserved proprioception in the perineum to cold, hot and painful stimuli. It is anticipated that the continence in them will improve with time primarily because of motivation and intact perineal sensation. Fortunately, we did not encounter any case with a high rectum necessitating a laparotomy. However, if the rectum is not found after opening the precoccygeal fascia, the patient can be turned supine and a laparotomy or laparoscopic mobilization of colon can be performed to gain sufficient length to proceed for a SSARP.

## Conclusion

Recently, authors have popularized exclusive laparoscopically assisted anorectal pull-through (LAARP) to reduce the amount of posterior dissection required for accurate placement of the bowel into the muscle complex [17,18]. In comparison to the above described technique, the step in the laparoscopic procedure of passage of the trocar through the perineum has the potential of injuring the urinary system. In addition, the incidence of postoperative prolapse is not yet known but may be a concern because of the avoidance of several key PSARP steps, most notably tacking of the rectum to the pelvic muscles [19]. Although the early results of SSARP are encouraging, long-term functional outcome of these patients are awaited. In conclusion, sphincter saving anorectoplasty (SSARP) allows safe, minimally invasive and anatomical reconstruction of the anorectum with a satisfactory function and cosmetic outcome.

## Competing interests

The author(s) declare that they have no competing interests.

## Authors' contributions

AP conceived the technical innovation and performed the surgery. AK, SA, RB and BM contributed towards refining the technique. AT provided radiographic imaging support. SNS helped in conducting surgery. BHP conducted electromyographic studies. All Authors read and approved the final manuscript.

## Pre-publication history

The pre-publication history for this paper can be accessed here:


